# Comparison of Nicotine and Selected Flavoring Contents Between Tobacco-Free and Tobacco-Containing Oral Pouches

**DOI:** 10.1093/ntr/ntaf105

**Published:** 2025-05-19

**Authors:** Michelle K Page, Noel J Leigh, Ashleigh, C Block, Poppy H Marrano, Matthew B Travers, Juan E Adrover, Grace E Maley, Lauren A Koenig, Eman M Salem, Scott D Heldwein, Maciej L Goniewicz

**Affiliations:** Department of Health Behavior, Roswell Park Comprehensive Cancer Center, Buffalo, NY, USA; Department of Health Behavior, Roswell Park Comprehensive Cancer Center, Buffalo, NY, USA; Department of Health Behavior, Roswell Park Comprehensive Cancer Center, Buffalo, NY, USA; Department of Health Behavior, Roswell Park Comprehensive Cancer Center, Buffalo, NY, USA; Department of Health Behavior, Roswell Park Comprehensive Cancer Center, Buffalo, NY, USA; Department of Health Behavior, Roswell Park Comprehensive Cancer Center, Buffalo, NY, USA; Department of Health Behavior, Roswell Park Comprehensive Cancer Center, Buffalo, NY, USA; Department of Health Behavior, Roswell Park Comprehensive Cancer Center, Buffalo, NY, USA; Department of Health Behavior, Roswell Park Comprehensive Cancer Center, Buffalo, NY, USA; Department of Health Behavior, Roswell Park Comprehensive Cancer Center, Buffalo, NY, USA

## Abstract

**Introduction:**

Flavored oral tobacco-free pouches (ONPs) are novel products that resemble traditional oral tobacco-containing pouches (OTPs) in design and route of administration. However, ONPs contain nicotine, various additives, and a filler material rather than tobacco leaves. Our study compares nicotine content, release, and form, along with selected flavoring content, between brands of ONPs and OTPs.

**Methods:**

A convenience sample of flavored ONPs (*n* = 10 brands) and OTPs (*n* = 7 brands) was purchased between 2021 and 2023. Total nicotine content in the pouches, nicotine released over 1 hour, isomer ratio (R:S-nicotine), and 33 flavoring chemicals were measured using chromatography methods. Nicotine form (the proportion of nicotine in the protonated versus freebase forms) was calculated after measuring the pH.

**Results:**

Although ONPs contained, on average, 50% less nicotine than OTPs (6.4 ± 3.5 vs. 12.3 ± 8.2 mg/pouch, *p* < .001), they released similar amounts of nicotine within 5 minutes (ONPs 6.5 ± 3.9 vs. OTPs 7.6 ± 3.9 mg/pouch, *p* = .422). The nicotine used in ONPs compared to OTPs was primarily freebase (63.4%±25.2% vs. 47.2%±34.4%, *p* = .223). Two ONPs contained racemic nicotine, suggesting their synthetic source. Menthol was the most frequently used flavoring chemical in both ONPs and OTPs. Triacetin was the most concentrated flavoring in ONPs (12.7 ± 12.7 mg/pouch) and methyl salicylate the most in OTPs (25.1 ± 1.8 mg/pouch).

**Conclusions:**

ONPs have a similar nicotine release profile as OTPs but contain a high fraction of the freebase nicotine and a wide range of flavorings. The presence of freebase nicotine and flavorings in ONPs may contribute to differential abuse liability of those novel products compared to OTPs.

**Implications:**

This is the first non-industry study to compare nicotine content, release, form, and select flavoring composition between multiple ONPs and OTPs. These findings have implications if any future modified risk claims for ONPs are considered. Although nicotine content was significantly higher in OTPs than in ONPs, the amount of nicotine released from the pouch was very similar between ONPs and OTPs. While many flavoring chemicals present in ONPs and OTPs are generally recognized as safe for oral consumption, their potential oral health risk in ONP and OTP users has not been evaluated and therefore must be monitored and assessed.

## Introduction

Noncombustible commercial tobacco products have evolved rapidly over the past two decades. The latest iteration, tobacco-free oral nicotine products, includes nicotine-containing pouches, lozenges, gummies, and chewing gums. Unlike electronic cigarettes, most oral tobacco-free pouches (referred to as ONPs) and, similarly, conventional oral tobacco-containing pouches (referred to as OTPs) are manufactured by the five largest tobacco companies rather than independent businesses.^1^ Flavored ONPs have been commercially available since 2016,^2^ and their use has increased by at least 300%.^3^ This increase may be partially attributed to the aggressive marketing of these products as tobacco-free alternatives to conventional tobacco products. The industry has previously used similar marketing strategies to alleviate consumers’ health concerns.^4^ However, the popularity of contemporary oral products has also been accredited to the availability of flavorings, including fruit, sweet, cooling, and tobacco flavors, similar to electronic cigarettes.^5^ In contrast, pre-portioned OTPs like snus have been commercially available since the 1970s in the United States,^6,7^ with a much slower consumer uptake.^8^ Although OTPs contain processed tobacco leaf, these products are perceived as less harmful compared to cigarettes^9^ because of lack of combustion and inhalation exposure. Several OTPs have received Modified Risk Granted Orders by the US Food and Drug Administration (FDA), which have demonstrated a significant reduction in risk and harm of tobacco-related diseases relative to combustible use.^10^ In January 2025, 20 flavored ONPs from ZYN received Marketing Granted Orders by the FDA, demonstrating sufficient evidence of greater benefit than risk to public health relative to combustible tobacco and OTP use.^11^

Both ONPs and OTPs are designed to deliver nicotine to users. All pouches must be placed between the gum and lip to promote salivation and nicotine release.^1^ The doses of nicotine delivered from the pouch to the user depend on multiple factors, including the total nicotine content in the pouch and the fraction of nicotine effectively released from the pouch into saliva. The speed of nicotine delivery from the pouch to the user also depends on multiple variables, including the protonation of nicotine molecules, where an increased proportion of nicotine in its freebase form (unprotonated) improves absorption by buccal mucosa,^12^ and the rate at which nicotine is released from these products. Few studies have examined the nicotine content and release from various ONPs^13,14^; however, no comprehensive comparison of nicotine characteristics and flavoring content between various ONP brands or OTPs has been made.

A recent review found that only five studies have compared toxicant levels between ONPs and OTPs; two of which have been funded by the industry.^15^ Tobacco-specific nitrosamine (TSNA) concentrations in ONPs are significantly reduced compared to OTPs.^16^ Lower levels of heavy metals, such as lead and arsenic, and mycotoxin (ochratoxin A) were found in one ONP brand compared to OTPs.^17^ Most ONPs contained lower amounts of formaldehyde and acetaldehyde compared to OTPs.^18^ However, only a few studies have examined other chemicals in ONPs, including flavorings. Although ONPs do not put users at risk for inhalation-related harm, some highly concentrated flavoring agents have been shown to cause acute oral toxicity in mice and rats.^19,20^ In addition, previous in vitro studies have shown that flavored ONPs may be cytotoxic to human gingival cells.^21,22^ Thus, it is essential to examine the total chemical composition of ONPs to adequately assess the potential health consequences of using those products.

This study compared nicotine concentration, release, form, and selected flavoring contents between a convenience sample of ONP and OTP popular brands available for sale in the United States. It also compared physical characteristics such as pouch weights and product packaging, including reported ingredients and warning labels.

## Methods

### Product Selection and Initial Evaluation

A convenience sample of 50 ONPs was selected from a local distributor in Buffalo, NY, or via online vendor searches for available ONP brands, without restrictions on flavor descriptors. This included 10 brands that were popular online at the time of the study initiation (BRIDGE, FRĒ, Juice Head, LUCY, on!, NIC-S, Rogue, VELO, White Fox, ZYN). Those selected brands were purchased with 25 unique flavor descriptors and labeled nicotine content ranging from 3 to 16 mg. One ONP brand (VELO) was purchased in different package types (metal tin and plastic can), while a second brand (LUCY) was purchased with and without an additional flavor capsule (“kapsel”) added to the pouch. Those ONPs were compared to 11 OTPs from seven brands (Bull Dog, Camel, Grizzly, General, Siberia, Skoal, Copenhagen) with nine unique flavors purchased based on availability from the same distributor, or via web searches for available OTPs. Except for one brand (Bull Dog), the nicotine content of OTPs was not indicated on package labeling. A complete list of purchased brands and their labeled nicotine strengths and flavors is reported in Table S1, and product images are available in Figure S1. All products were purchased between March 2021 and April 2023 and stored at 4 ± 2°C in a dark place until analysis. The CORESTA smokeless tobacco reference product (CRP1.1 Swedish-style snus pouches) was included as an analytical control.

Three pouches were randomly selected from each pack, and the average weight (filler material and pouch wrapper) was determined using an analytical balance (Mettler Toledo) with an accuracy of ±0.0001 g. The average weight was compared between ONPs and OTPs. Package labeling was visually inspected, and web searches were conducted to identify pouch ingredients reported by manufacturers. Manufacturer-owned websites were prioritized (70%), followed by vendor websites (30%) as a proxy when manufacturer sites were unavailable. Warning labels on product packages were identified and evaluated for content following the previously reported methodology.^23^ We coded legal disclaimers, statements related to health outcomes, and nicotine content. The frequency and content of warning labels were compared between ONPs and OTPs.

### Nicotine Characteristics

#### Nicotine Content

A single pre-weighed ONP or OTP was vortexed for 15 minutes in 20 mL of a methanol extraction solution containing 25 µg/mL of internal standard (naphthalene-d8), then filtered with a 0.45 µm polytetrafluoroethylene syringe filter. The extract was diluted 10-fold with the extraction solution and analyzed using a gas chromatography/mass spectrometry (GC/MS) method using parameters described previously.^24^ Calibration curve concentrations for nicotine were between 1.0 and 200.0 µg/mL, corresponding to nicotine content in a pouch between 0.2 and 40 mg/pouch (lower limit of quantitation of 0.2 mg/pouch). All products were prepared in triplicate, and average values from three tests are reported. For ONPs, the values of measured and labeled nicotine contents were compared and reported as relative differences. Content per pouch and gram of pouch was also compared between ONPs and OTPs.

#### Nicotine Release

In addition to determining nicotine content in the products, we also aimed to compare how much nicotine can be released from both product types and how fast it can be released. The rate of nicotine released from each pouch was determined once per brand and strength (*n* = 50 ONPs; *n* = 11 OTPs) over 1 hour. To compare products systematically, we used an experimental approach to facilitate maximal and more rapid nicotine release from products, rather than replicate human oral use. Milli-Q water (10 mL) was pre-heated to 37°C in a 50-mL glass beaker using a magnetic hot plate. A magnetic stir bar was set to 350 rpm. A single pouch was added, and aliquots (50 µL) were taken initially (before adding the pouch) and every 5 minutes up to 60 minutes. After diluting aliquots 200-fold with methanol and internal standard solution (5 µg/mL, rac-nicotine-d4), samples collected at 0, 5, 10, 15, 30, and 60 minutes were analyzed for each product. Samples were analyzed using a Shimadzu Nextera X2 LC system and a Sciex 6500+ tandem mass spectrometer (LC-MS/MS) with an AZYP NicoShell chiral column, following settings previously described.^25^ Calibration curves ranged from 0.01 to 7.5 µg/mL, corresponding to 0.02–7.5 mg/pouch of nicotine (LLOQ of 0.02 mg/pouch). If measured concentrations exceeded the calibration range, the samples were diluted 2- or 4-fold and re-analyzed. We report the concentration of nicotine and the percentage of total nicotine content (as determined with the GC/MS assay) released after each time point. If the measured release exceeded the measured total content due to instrument variation, the released percentage was 100%. The nicotine amount and percentage of nicotine content in a pouch released after 5 minutes were compared between ONPs and OTPs.

#### Nicotine Isomers

Using the LC-MS/MS method described above, we determined the presence of R- and S-nicotine isomers in the samples collected during nicotine release tests. Equal amounts of R- and S-isomers (R,S-racemic) were a potential indicator of synthetic nicotine. Pouches containing more than 90% S-nicotine were suspected to contain nicotine derived from tobacco.

#### Nicotine Form (Percent Freebase)

A pre-weighed pouch was mixed for up to 30 minutes in 10 mL Milli-Q water, following a modified version of CORESTA Recommended Method 69.^26^ After equilibrating to room temperature, the pH was measured using a Mettler Toledo Seven Compact pH meter. The percentage of nicotine in the unprotonated (freebase) form was calculated using the Henderson–Hasselbalch equation (using a nicotine pKa of 8.01). The average percentage of freebase nicotine was compared between ONPs and OTPs.

### Flavoring Content

We analyzed pouches for the content of 33 flavoring chemicals commonly used in tobacco products, including 1,4-cineole, 2,3,5-trimethylpyrazine, acetoin, benzaldehyde, benzyl alcohol, butanoic acid, carvone, cinnamaldehyde, dihydroxyacetone, ethyl maltol, ethyl salicylate, ethyl vanillin, eucalyptol, eugenol, fenchol, furaneol, isomenthol, isopulegol, isovanillin, limonene, linalool, maltol, menthol, menthone, menthyl acetate, methyl salicylate, piperitone, pulegone, raspberry ketone, triacetin, vanillin, WS-3, and WS-23, following a GC/MS method previously developed to measure these same flavorings in cigarettes.^24^ Pouches were extracted in the same methanol extraction solution described above for the total nicotine content assay. Calibration curves ranged from 0.25 to 100 µg/mL, corresponding to 0.05–20 mg/pouch (LLOQs, Table S2). If measured concentrations exceeded the calibration range, the samples were diluted 10-fold and re-analyzed. Additionally, since each pouch of LUCY brand (Cool Cider, Spearmint, Mango) contained one flavor capsule, the capsule was removed from the pouch and crushed to release the inner material. Analysis of capsule content was performed following previously reported methodology.^27,28^ Frequency and average concentrations of detected flavorings were compared between ONPs and OTPs.

### Statistical Analysis

Statistical analysis was performed using Prism GraphPad version 10.3.1. We used the Mann–Whitney nonparametric *t*-test to compare nicotine characteristics, pH, pouch weight, and flavoring content between ONPs and OTPs.

## Results

### Product Weight

ONP weights ranged from 0.3 ± 0.0 g (on!) to 0.6 ± 0.0 g (Rogue), while OTPs ranged from 0.6 ± 0.1 g (Camel) to 1.6 ± 0.1 g (Copenhagen; Table S1). On average, ONPs were significantly lighter than OTPs (0.5 ± 0.1 vs. 0.9 ± 0.4 g, *p* < .001).

### Product Characteristics as Reported by Manufacturers

Product packaging and content analysis on manufacturer websites revealed that most ONP brands contained cellulose-based filler material (80%, Table S3). The primary filler material reported in all OTPs was tobacco. ONP brands LUCY, NIC-S, and Rogue disclosed nicotine polacrilex, where nicotine is bound to an ion exchange resin and delivered via an aqueous extraction, similar to therapeutic nicotine gums.^29^ ZYN and on! further indicated that nicotine in these products was salt-based. ZYN specifically disclosed the use of nicotine bitartrate dihydrate. Several additional descriptors were used to describe the source of nicotine in ONPs, such as “non-tobacco nicotine” (BRIDGE, FRĒ) and “zero-tobacco synthetic nicotine” (Juice Head). Labeling of ONPs indicated the presence of artificial sweeteners such as acesulfame potassium (Ace K) and sucralose (Table S3). OTP manufacturers also reported using sweeteners such as Ace K, sucralose, and sodium saccharin. Natural and artificial flavorings were listed for ONPs and OTPs. Nearly all ONP manufacturers acknowledged using chemicals to modify pouch pH, most commonly sodium bicarbonate and sodium carbonate (Table S3). A wider variety of pH adjusters were also reported in OTPs, which included ammonium-, magnesium-, potassium-, and sodium carbonates. All ONP brands except White Fox specified use duration; the instructions ranged from 15 to 60 minutes (Table S3). In contrast, only Camel, General, and Siberia provided recommended durations for product use (ranging from 30 to 45 minutes).

Warning labels on ONPs referred only to their nicotine content; all ONPs contained a warning that they “contain nicotine” and “nicotine is addictive” (Table S4). Most ONPs (70%) referred to California’s Proposition 65, a list of chemicals known to cause cancer, birth defects, or other reproductive harm, in smaller text. VELO specifically stated exposure to acrylamide (tins) and acetaldehyde (cans). In contrast, OTPs did not specify whether the product contained nicotine, although over half the brands claimed to be addictive. Warnings for gum disease and tooth loss were present in four OTP brands, followed by mouth cancer and nonspecified damage (each in two OTP brands). Four OTP brands also included a warning that they were not a safe alternative to combustible cigarettes.

### Measured Nicotine Characteristics

**Figure 1.**
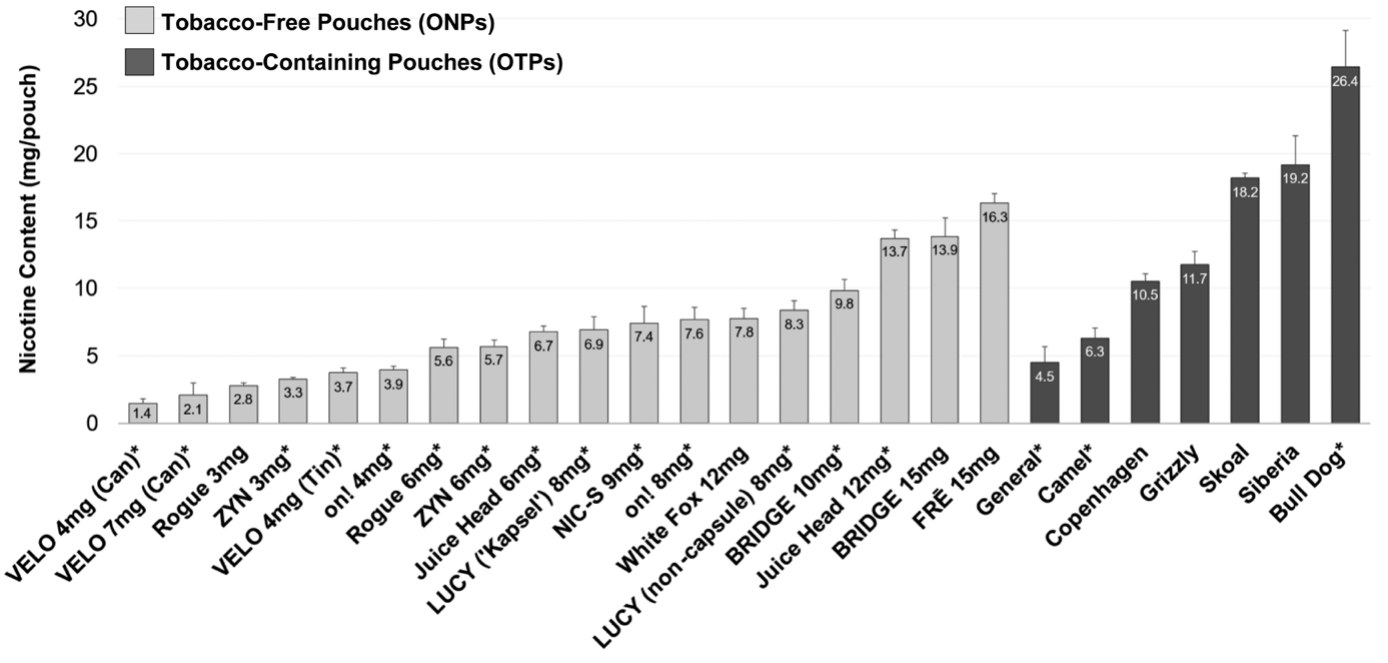
Measured nicotine content by brand and labeled nicotine strength for oral tobacco-free pouches (ONPs) and oral tobacco-containing pouches (OTPs) used in this study. Error bars represent standard deviation; *indicates average and standard deviation among multiple flavors within a brand and nicotine strength. The average nicotine content in ONPs (6.4 ± 3.5 mg/pouch) was significantly lower than in OTPs (12.3 ± 8.2 mg/pouch) (*p* < .001; a nonparametric Mann–Whitney *t*-test).

Nicotine content in ONPs ranged from 1.4 ± 0.4 mg/pouch (VELO 4 mg in cans) to 16.3 ± 0.7 mg/pouch (FRĒ 15 mg). Nicotine in OTPs ranged from 4.5 ± 0.6 mg/pouch (General) to 26.4 ± 1.3 mg/pouch (Bull Dog, Figure 1, Table S1). Overall, OTPs contained more nicotine per pouch than ONPs (12.3 ± 8.2 vs. 6.4 ± 3.5mg/pouch, *p* < .001). However, per gram of pouch material, both ONPs and OTPs contained similar amounts of nicotine (14.6 ± 7.4 vs. 14.3 ± 8.2 mg/g, *p* = .487, Table S1). With similar numbers of pouches available per package (18 ± 3 vs. 18 ± 4, *p* = .771), the estimated total nicotine per gram in each package was not significantly different (259.7 ± 153.6 vs. 253.9 ± 185.9 mg/g, *p* = .487). Nicotine was not detected in any flavor capsule in LUCY products. For most ONPs, measured nicotine content was consistent with values provided on packages (within 20%; Table S1). VELO nicotine pouches available in 4 and 7-mg cans contained, on average, >60% less nicotine than labeled (1.4 ± 0.4 and 2.1 ± 0.9 mg/pouch, respectively; Table S1). Likewise, White Fox 12 mg contained nearly 40% less nicotine (7.8 ± 0.8 mg/pouch) than labeled. Only one OTP brand (Bull Dog) reported nicotine concentration on the packaging. Although strength was labeled as five out of six “paw prints,” online marketing reported as 22 mg, our analysis revealed Bull Dog contained 20.0% ± 5.8% (4.4 ± 1.3 mg/pouch) more nicotine (Table S1).

**Figure 2.**
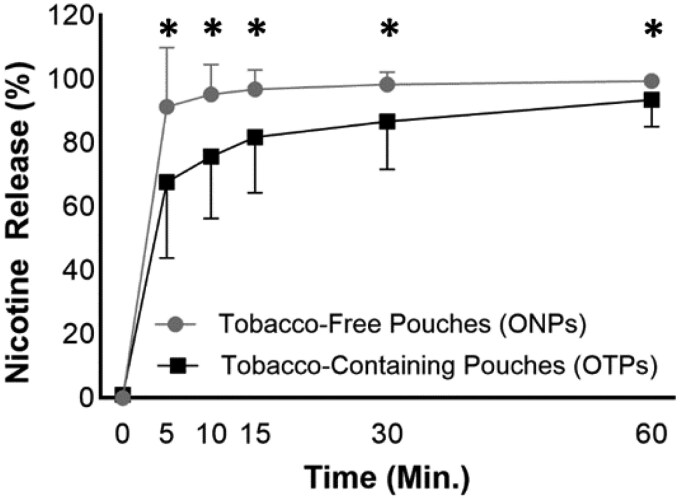
Average nicotine release among all oral tobacco-free pouches (ONPs) and oral tobacco-containing pouches (OTPs) used in this study. Error bars represent standard deviation. ^*^Indicates statistical significance (p < .05) between ONPs and OTPs using a nonparametric Mann–Whitney *t*-test.

Despite differences in nicotine content per pouch, ONPs released similar amounts of nicotine as OTPs within the first 5 minutes (6.5 ± 3.9 vs. 7.6 ± 3.9 mg/pouch, respectively, *p* = .422, Table S1). This resulted from a more significant proportion of total nicotine content released from ONPs than OTPs (Figure 2, Table S1). Among ONPs, most pouches released >80% of total nicotine content within 5 minutes (Table S1, Figure S2). A delayed release was observed from Rogue pouches, where spearmint 6-mg pouches released 72.3% of their nicotine content and wintergreen 3-mg pouches released <1% at 5 minutes (Figure S2). In addition, non-capsule LUCY 8 mg cinnamon and mango flavor products released their nicotine slower (36.6 and 42.4% at 5 minutes) than the non-capsule LUCY 8 mg wintergreen flavor (90.8%, Table S1). Among OTPs, only Copenhagen, General, and Grizzly released at least 80% of their nicotine content after 5 minutes. All nicotine was released from ONPs within 60 minutes, whereas only 55% of OTPs released all nicotine within 60 minutes (Figure S2).

Several ONP brands claimed to use tobacco-free nicotine; however, only BRIDGE and FRĒ pouches contained racemic nicotine (Table S1). The remaining ONPs and all OTPs contained 100% S-nicotine.

Average pH of ONP brands ranged from 7.0 (VELO 4 mg) to 9.0 (FRĒ 15 mg) (Table S1), which correspondingly ranged from 0.9% to 89.7% freebase nicotine. Inconsistencies in pH between BRIDGE pouches of different flavors were observed, where spearmint and cool mint flavors were more alkaline compared to wintergreen-flavored pouches (pH 9.0 vs. 6.0). Likewise, VELO 4- and 7-mg pouches from cans were more alkaline than from tins (8.4 vs. 7.0). pH of OTPs ranged from 6.8 (Copenhagen) to 9.4 (Siberia) (Table S1), which corresponded to 5.2% versus 96.0% freebase nicotine. Overall, ONPs contained a greater albeit insignificant proportion of freebase nicotine than OTPs (63.4% ± 25.2% vs. 47.2% ± 34.4%, *p* = .223).

### Measured Flavoring Content

**Figure 3.**
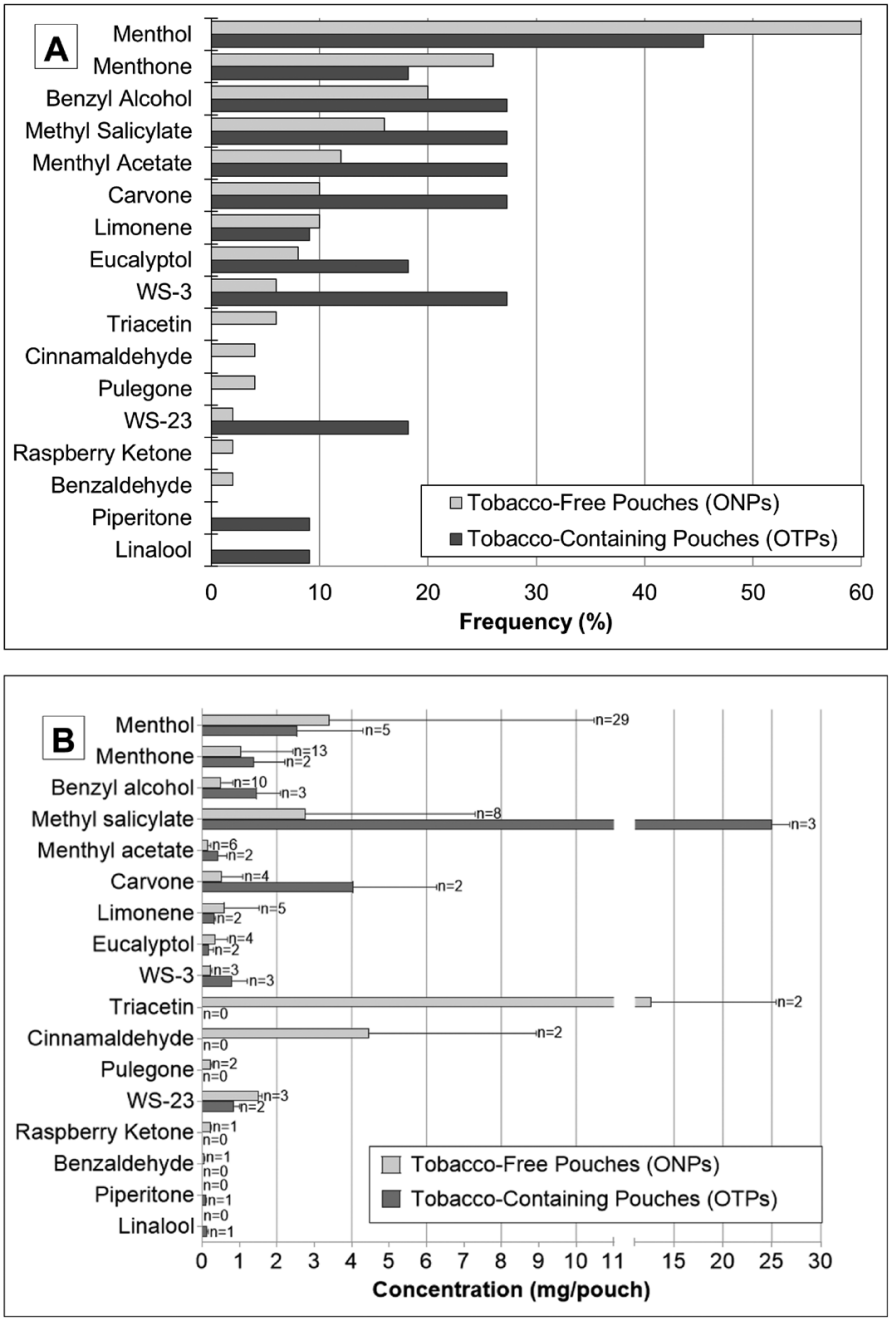
(**A**) Flavoring chemical frequency and (**B**) average concentration among products only with identified flavorings in oral tobacco-free pouches (ONPs) and oral tobacco-containing pouches (OTPs). *N* represents the number of products. Error bars represent standard deviation. ^*^Indicates statistical significance (*p* < .05) between ONPs and OTPs using a nonparametric Mann–Whitney *t*-test. Values with a single error bar (e.g., triacetin) could not be compared statistically.

Although a greater number of flavorings were detected in ONPs than OTPs overall (15 vs. 12, Figure 3A), OTPs contained more flavorings per pouch than ONPs (2.7 ± 2.8 vs. 1.9 ± 1.5, *p* = .919, Figure 3B, Table S6). The most common flavorings in ONPs were menthol (cooling) (60.0%), menthone (26.0%), benzyl alcohol (20.0%), and methyl salicylate (16.0%). Similarly, menthol was identified most frequently in OTPs (45.5%), followed by benzyl alcohol, methyl salicylate, menthyl acetate, carvone, and WS-3 (27.3% each). Menthol was identified in nearly every mint-flavored ONP and OTP across all tested brands (Table S5). Interestingly, more cooling flavorings, including synthetic cooling chemicals WS-3 and WS-23, were identified in OTPs than in ONPs. WS-3 was also the only ingredient identified in the single brand of ONP with an implicit flavor descriptor (ZYN 3 and 6 mg Chill). Expectedly, methyl salicylate, which gives a characteristic wintergreen taste, was identified in all ONP and OTP wintergreen flavors. Benzyl alcohol, which provides a fruity taste, was identified across ONPs with various flavor descriptors, including fruit, mint, and spice, along with three OTP brands with mint (General) and implicit flavor descriptors (Bull Dog Extreme and Cold Extreme).

The most concentrated flavorings identified in ONPs were triacetin (creamy) (Juice Head 12 mg Raspberry Lemonade Mint: 29.0 ± 0.1 mg/pouch), menthol (cooling) (Rogue Peppermint: 15.1 ± 1.2 mg/pouch), and methyl salicylate (wintergreen) (Rogue Wintergreen: 14.9 ± 0.5 mg/pouch) (Figure 3B, Table S6). On average, triacetin was the most concentrated flavoring across ONPs (12.7 ± 12.7 mg/pouch in three brands), while no OTP contained this flavoring (Figure 3). In OTPs, methyl salicylate was the most concentrated (Copenhagen Wintergreen: 26.8 ± 0.2mg/pouch), followed by carvone (minty) (Siberia Mint: 5.8 ± 2.3 mg/pouch) and menthol (Siberia Mint: 5.3 ± 0.4 mg/pouch). Methyl salicylate, on average, was the most concentrated across OTPs (25.0 ± 1.8 mg/pouch in three brands). Menthol and WS-23 (minty) were significantly higher in ONPs, whereas benzyl alcohol (fruity), carvone, menthyl acetate (cooling), methyl salicylate, and WS-3 (cooling) were considerably higher in OTPs (all *p* < .05).

Menthol was detected in flavor capsules in all three LUCY ONPs, ranging in concentration between 19.5 and 62.7 mg/capsule (Table S6). Four additional flavorings were identified in capsules: eucalyptol (minty) and limonene (citrus) in LUCY 8 mg Spearmint ranging from 0.1 to 3.5 mg/pouch, and pulegone (minty) in LUCY 8 mg Cool Cider and LUCY 8 mg Spearmint (0.2 and 0.3 mg/capsule, respectively).

## Discussion

This novel study compared chemical characteristics across a wide range of flavored ONPs and OTPs, including nicotine content, release, form, and selected flavoring contents. Overall, we found that ONPs are generally consistent with reported nicotine content. In contrast, of the two OTPs with reported nicotine strength, only one product was consistent (Bull Dog Extreme). However, our measured nicotine content in Camel and General products was consistent with prior studies.^30–32^ While ONPs contained roughly half the amount of nicotine per pouch compared to OTPs, they also weighed approximately half as much as OTPs. When considering nicotine delivery per gram of material and the number of total pouches per container, similar amounts of total nicotine are available for consumers, regardless of the product type. After 5 minutes, we observed similar nominal nicotine released from ONPs (6.5 mg/pouch) compared to OTPs (7.6 mg/pouch), despite significantly more rapidly released from ONPs (91.3%) than OTPs (67.8%). Delayed release among OTPs may offset the significantly higher nicotine content initially available in the product, since increased levels of nicotine may be more irritating to consumers. While we observed differences in the proportion of nicotine content released from ONPs and OTPs at 5 minutes, both product types released very similar nominal amounts of nicotine overall. Reported use of nicotine polacrilex by several brands may explain delayed nicotine release among several ONPs (e.g., Rogue 3 mg wintergreen, LUCY 8 mg cinnamon). However, future studies should substantiate these findings by using methods that replicate human use.

Several brands implied containing synthetic nicotine using terminology such as “non-tobacco nicotine.” Only two brands (BRIDGE and FRĒ) were confirmed to contain racemic (50:50 R:S) nicotine. Nicotine in other brands, such as Juice Head and NIC-S, which used similar descriptors, was found predominantly in the S-isomer form. While purified synthetic S-nicotine is possible, the cost is reportedly much higher than that of producing racemic synthetic nicotine.^33^ Carbon isotope methodologies can confirm whether these products contained synthetic S-nicotine. Nevertheless, using such terminology can be misleading to consumers, as it may not be clear whether “tobacco-free nicotine” or “non-tobacco nicotine” refers to the synthetic nicotine (lab-made) or simply a pouch devoid of tobacco material, regardless of the nicotine source.

While the nicotine form ranged from highly protonated (nearly 0% freebase) to almost wholly freebase among both ONPs and OTPs, we estimated, on average, ONPs contained higher proportions of freebase nicotine than OTPs, similar to previously reported findings.^34^ However, the differences across all tested products were not significant. The buccal mucosa in the mouth better absorbs freebase nicotine than highly protonated nicotine.^35^ Therefore, ONPs with a higher percentage of freebase nicotine are likely to deliver nicotine more effectively than some OTPs, which may increase their abuse liability potential compared to OTPs. Alternatively, an increasing proportion of freebase nicotine is associated with more irritation, and so duration of use will also impact nicotine delivery and abuse potential. Therefore, future pharmacokinetics and sensory studies with people who use pouches regularly are needed to compare the speed of nicotine delivery from OTPs and ONPs.

Since recent findings suggest an enhanced addiction potential of flavored oral tobacco-containing products (independent of nicotine), where flavors are associated with cognitive resource allocation, which sustains subjective experience after use,^36^ analyzing flavor availability in ONPs is critical to assess the abuse liability potential of those products. Although we used a convenience sampling from the US market rather than a survey approach, many more flavors were available in ONPs than in OTPs. Since flavors in both products are not currently regulated by The Family Smoking Prevention and Tobacco Control Act, it is interesting that OTP manufacturers provide a limited selection of flavors in the United States, where most are tobacco or menthol/mint, while providing more diverse flavors in their ONP product lines (e.g., General vs. ZYN, both owned by Swedish Match/ Phillip Morris International).

We identified menthol as an ingredient in mint/cooling-flavored ONPs and OTPs, yet also in fruit-flavored ONPs. Like e-cigarettes and conventional cigarettes, menthol may provide a cooling sensation even among products without explicit mint/cooling claims, to mask the harshness of nicotine, especially among those products with a high proportion of freebase nicotine. We also found synthetic coolants WS-23 in minty OTP flavors and WS-3 in minty ONP and OTP flavors. WS-3 was also present in the implicit flavor “Chill” from ZYN 3 and 6 mg, consistent with previous findings (0.3 ± 0.1 vs. reported 0.2 ± 0.0 mg/pouch).^37^ However, the measured concentration of other flavorings (menthol, carvone, menthyl acetate, and menthone) across ZYN flavors in our study was lower than previously reported (e.g., menthol: 0.9 ± 0.1 vs. reported 1.4 mg/pouch in ZYN menthol),^37^ likely given the difference in extraction time (15 minutes vs. 7 days) or from variations between ONP batches. Hybrid flavored ONPs, which were considered those with a mint/cooling descriptor and at least one additional flavor (e.g., “Mango Strawberry Mint”), each included menthol as an ingredient. One ONP hybrid flavor (LUCY 8 mg Cool Cider), which combined a beverage flavor with mint, also included menthol and WS-23, similar to hybrid flavored e-cigarettes.^38^ Interestingly, neither synthetic coolant was identified in ONPs with flavors other than mint/cooling. This differs from e-cigarettes, where WS-23 has been identified in fruity flavors without explicit cooling descriptors.^25^ Likewise, fewer synthetic coolants were found in mint/cooling ONPs than in similarly flavored OTPs.

Triacetin, which has a creamy flavor and is frequently added to e-cigarette liquids,^27^ was found to be the most concentrated flavoring chemical detected in three ONPs but was not detected in any OTPs. While triacetin is a commonly added plasticizer in other tobacco products,^39,40^ its role in ONPs remains unclear.

Our detected levels of flavorings in OTPs were similar to those measured previously in smokeless tobacco products (e.g., average methyl salicylate: 17.1 ± 0.6 vs. reported 21.9 mg/g among wintergreen flavors).^41^ Concerningly, pulegone, a suspected human carcinogen banned as a food additive by the FDA,^42^ was measured in two capsules added to LUCY pouches. Previous findings have shown increased cytotoxicity of ONPs in oral cell line models observed across different flavors and nicotine content.^21,22^ Our results and previous findings^21,22^ have implications for potential adverse health effects of flavored ONP use, warranting further investigation.

A limitation of our study is that a convenience sample was used to compare ONPs to OTPs. As such, nearly five times more ONPs than OTPs were included in analyses, and observed differences may be explained by discrepancies in the sample size. For example, synthetic coolants were found less frequently among ONPs than OTPs, yet this finding is likely explained by oversampling mint/cooling flavors among OTPs. These results, therefore, should only be used to make broad inferences about differences between these product types, as they did not represent all ONPs and OTPs at the time of purchase. The availability or design of the products may have changed since they were purchased. For example, LUCY pouches with capsules, which were labeled as “kapsels” are now branded as LUCY “breakers.” Likewise, we conducted measurements within the same container, so variability between containers was not assessed in our study. It would be helpful to re-examine the same products between containers and determine whether physical, chemical, or marketing changes have occurred over time. Another limitation of this study is that our nicotine release method was not designed to replicate human use, similar to established industry methodologies,.^13,43^ Instead, our systematic approach facilitated the release of nicotine as a means to compare relative availability between product types. For example, pouches that release nicotine slower likely indicate less nicotine is immediately available for consumers compared to pouches with a rapid nicotine release. Because of this, our nicotine release rates cannot be compared to those from human studies. Another limitation is that we only examined the release every 5 minutes. Earlier and more frequent time points (e.g., every 1min for the first 5 minutes) might have captured more information about rapid nicotine release from ONPs.

Some limitations of our analytical assays also need to be considered. We did not adjust the pH during nicotine extraction, as reported by other published methods.^34^ Nicotine results, therefore, may be underreported. Nonetheless, as measured by our method, nicotine content in the CORESTA reference product CRP1.1 was within 20% of previously reported content^44,45^ (Table S1). Among all tested products (*n* = 61), 18% (*n* = 11) showed higher nicotine release by more than 15% as measured by the LC-MS/MS assay compared to the total content detected by the GC/MS assay. This may be related to potential interactions between the filler material or other additives (e.g., flavorings, humectants) that may differentially interact with extraction solvents (water used in the LC-MS/MS assay vs. methanol used in the GC/MS assay). For example, we observed thickening of the water extraction solution with some products over 1 hour. These potential interactions between filler material and additives in the pouch require further investigation.

In the ONP brand VELO sold in cans, nicotine was nearly 70% lower than the label when analyzed using the GC/MS assay. However, the measured nicotine released at 60 minutes was similar to the label, suggesting that methanol did not efficiently extract nicotine from this formulation. Interestingly, both VELO flavors purchased in tins had comparable measured nicotine content to the label. VELO specified different materials used for the pouch filler; those available in tins reportedly contain microcrystalline cellulose, whereas those in cans claim to use modified cellulose. Modified cellulose is an umbrella term encompassing various cellulose derivatives used in food products, including microcrystalline cellulose.^46^ It is unclear what form of modified cellulose is added to VELO pouches available in cans; however, this finding suggests a different material is used in the product of the same brand sold in tins. Our results indicate that chemical characteristics of ONPs may differ not only across brands, but even within the same brand.

Despite the variety of flavors, we detected relatively few of our targeted flavoring chemicals, particularly across ONPs. The assay did not include alternative ingredients, such as ethyl butyrate, specific to artificial flavors like mango. The targeted list of ingredients was based on frequencies previously identified from studies of similarly flavored e-cigarettes.^47^ Additionally, we did not measure other additives, such as artificial sweeteners. ONP and OTP manufacturers reported using Ace K, sucralose, and sodium saccharin. Such artificial sweeteners are reportedly 600 times sweeter than natural sugar.^48^ We confirmed manufacturers were not adding natural sugars using a Sucrose/D-Fructose/D-Glucose assay (Neogen, Lansing, MI) on select ONP (VELO, NIC-S, and on!) and OTP (Camel and General) brands (data not shown). Prior data have identified sucralose as high as 11 mg/pouch in OTPs.^49^ Future work should consider evaluating the content of sweeteners and other flavoring ingredients added to ONPs and their impact on product use and health risk.

## Conclusions

ONPs are a novel class of recreational nicotine products that release similar amounts of nicotine as OTPs. Some ONP brands had high proportions of nicotine in freebase form, which may facilitate buccal absorption, leading to a greater abuse liability. ONPs contain more flavoring chemicals, comparatively, some in high concentrations, and more work is needed to understand potential toxicity. The availability of flavors in ONPs (beyond menthol and mint) may increase the initiation of ONP use and potentially affect the harmfulness of the products.

## Supplementary Material

ntaf105_suppl_Supplementary_Tables_S1-S5_Figures_S1-S2

ntaf105_suppl_Supplementary_Table_S6

## Data Availability

The corresponding author will share the data underlying this article at a reasonable request.
