# Supplementary material for: Comparison of Nicotine and Selected Flavoring Contents Between Tobacco-Free and Tobacco-Containing Oral Pouches
Source: Nicotine Tob Res. 2025 May 19;28(2):268–76. doi: 10.1093/ntr/ntaf105 (PMC12824957; doi:10.1093/ntr/ntaf105)
Supplement: ntaf105_suppl_Supplementary_Tables_S1-S5_Figures_S1-S2 [file ntaf105_suppl_supplementary_tables_s1-s5_figures_s1-s2.docx]

**SUPPLEMENTAL MATERIALS**

**Supplemental Table 1**. Nicotine characteristics of oral tobacco-free pouches (ONPs) and oral tobacco-containing pouches (OTPs). ^1^Indicates statistical significance (*p*<0.05) between ONPs and OTPs using a non-parametric Mann-Whitney t-test. ^2^The package used the "Paw Prints" scale to indicate concentration, which could be compared to online claims.

| **Brand** | **Flavor** | **Labeled Nicotine Strength (mg)** | **Pouch Weight  (g)** | **Measured Nicotine Content (mg/**  **pouch)** | **Measured Difference from Labeled Content  (%)** | **Estimated Nicotine Content (mg/g of pouch)** | **Proportion of Nicotine Released at 5 mins (%)** | **Measured Nicotine Released at 5 mins (mg/**  **pouch)** | **S:R**  **Nicotine Ratio** | **pH** | **% Free**  **base** |
| --- | --- | --- | --- | --- | --- | --- | --- | --- | --- | --- | --- |
| **Tobacco-Free Pouches (ONPs)** | | | | | | | | | | | |
| BRIDGE | Spearmint | 10 | 0.5±0.0 | 9.5±0.6 | -5.2 | 19.1 | 100 | 11.3 | 50:50 | 9.1 | 91.6 |
|  | Wintergreen | 10 | 0.5±0.0 | 10.2±1.0 | 1.7 | 22.2 | 79.4 | 8.1 | 50:50 | 6.0 | 0.9 |
|  | Cool Mint | 15 | 0.5±0.0 | 13.9±1.3 | -7.6 | 28.6 | 96.6 | 13.4 | 50:50 | 9.0 | 90.5 |
| FRĒ | Mint | 15 | 0.5±0.0 | 16.3±0.7 | 8.7 | 31.6 | 87.5 | 14.3 | 50:50 | 9.0 | 89.7 |
| Juice Head | Mango Strawberry Mint | 6 | 0.6±0.0 | 6.5±0.3 | 8.6 | 11.3 | 100 | 6.9 | 100:0 | 7.4 | 21.2 |
|  | Watermelon Strawberry Mint | 6 | 0.6±0.0 | 7.0±0.4 | 16.4 | 11.7 | 100 | 8.1 | 100:0 | 7.3 | 15.7 |
|  | Blueberry Lemon  Mint | 12 | 0.5±0.0 | 13.1±0.1 | 9.3 | 24.0 | 80.6 | 10.6 | 100:0 | 7.4 | 18.6 |
|  | Peach Pineapple Mint | 12 | 0.6±0.0 | 13.9±0.4 | 15.4 | 23.7 | 94.1 | 13.1 | 100:0 | 7.8 | 37.1 |
|  | Raspberry Lemonade Mint | 12 | 0.7±0.0 | 14.2±0.4 | 18.5 | 20.9 | 95.6 | 13.6 | 100:0 | 7.2 | 12.4 |
| LUCY (Non-Capsule) | Cinnamon | 8 | 0.6±0.0 | 8.1±1.1 | 1.3 | 14.6 | 36.6 | 3.0 | 100:0 | 8.4 | 72.5 |
|  | Mango | 8 | 0.6±0.0 | 8.5±0.4 | 6.8 | 14.9 | 42.4 | 3.6 | 100:0 | 8.3 | 65.6 |
|  | Wintergreen | 8 | 0.6±0.0 | 8.4±0.7 | 5.0 | 15.0 | 90.8 | 7.6 | 100:0 | 8.5 | 73.4 |
| LUCY "Kapsel" | Cool Cider | 8 | 0.5±0.0 | 5.7±0.3 | -28.5 | 12.1 | 80.2 | 4.6 | 100:0 | 8.4 | 72.0 |
|  | Mango | 8 | 0.4±0.0 | 7.8±0.4 | -2.4 | 20.7 | 82.7 | 6.4 | 100:0 | 8.2 | 59.7 |
|  | Spearmint | 8 | 0.3±0.0 | 7.2±0.2 | -10.3 | 21.2 | 93.9 | 6.7 | 100:0 | 8.5 | 76.0 |
| NIC-S | Mixed Berry | 9 | 0.5±0.0 | 7.7±0.2 | -14.3 | 16.3 | 100 | 8.7 | 100:0 | 8.5 | 75.6 |
|  | Spiced Cinnamon | 9 | 0.5±0.0 | 7.1±1.7 | -20.7 | 14.3 | 100 | 7.3 | 100:0 | 8.7 | 83.4 |
|  | Sweet Orange | 9 | 0.5±0.0 | 7.5±1.6 | -17.1 | 15.7 | 93.7 | 7.0 | 100:0 | 8.7 | 81.7 |
|  | Wintergreen | 9 | 0.4±0.0 | 7.5±1.1 | -16.8 | 17.3 | 100 | 22.2 | 100:0 | 8.7 | 84.0 |
| on! | Mint | 4 | 0.3±0.0 | 3.9±0.4 | -2.7 | 14.2 | 99.6 | 3.9 | 100:0 | 9.3 | 95.2 |
|  | Original | 4 | 0.3±0.0 | 3.9±0.5 | -2.0 | 14.3 | 93.9 | 3.7 | 100:0 | 8.4 | 69.1 |
|  | Wintergreen | 4 | 0.3±0.0 | 3.9±0.2 | -1.9 | 14.8 | 100 | 4.3 | 100:0 | 9.3 | 94.8 |
|  | Berry | 8 | 0.3±0.0 | 7.7±0.7 | -4.2 | 27.0 | 100 | 8.1 | 100:0 | 8.4 | 68.6 |
|  | Cinnamon | 8 | 0.3±0.0 | 6.8±0.6 | -15.1 | 26.4 | 100 | 8.2 | 100:0 | 9.2 | 94.2 |
|  | Citrus | 8 | 0.3±0.0 | 7.9±0.8 | -1.6 | 28.4 | 100 | 8.6 | 100:0 | 8.4 | 70.1 |
|  | Coffee | 8 | 0.3±0.0 | 8.3±1.1 | 3.4 | 30.3 | 85.3 | 7.1 | 100:0 | 8.4 | 71.5 |
| Rogue | Wintergreen | 3 | 0.6±0.0 | 2.8±1.9 | -6.5 | 4.6 | 0.7 | 0.0 | 100:0 | 7.9 | 42.6 |
|  | Peppermint | 6 | 0.6±0.0 | 5.9±0.7 | -1.3 | 10.1 | 90.1 | 5.3 | 100:0 | 8.0 | 48.3 |
|  | Spearmint | 6 | 0.6±0.0 | 5.3±0.6 | -11.3 | 8.4 | 72.3 | 3.8 | 100:0 | 7.8 | 37.1 |
| VELO (Tin) | Citrus | 4 | 0.4±0.0 | 3.8±0.2 | -4.6 | 8.9 | 100 | 4.6 | 100:0 | 7.0 | 9.1 |
|  | Mint | 4 | 0.4±0.0 | 3.7±0.5 | -8.6 | 8.5 | 100 | 4.0 | 100:0 | 7.0 | 8.7 |
| VELO (Can) | Coffee | 4 | 0.4±0.0 | 1.1±0.1 | -71.3 | 2.9 | 100 | 2.1 | 100:0 | 7.8 | 40.3 |
|  | Peppermint | 4 | 0.4±0.0 | 1.4±0.1 | -64.9 | 3.8 | 100 | 3.8 | 100:0 | 8.0 | 51.7 |
|  | Spearmint | 4 | 0.4±0.0 | 1.8±0.1 | -54.3 | 4.9 | 100 | 2.2 | 100:0 | 9.0 | 91.3 |
|  | Black Cherry | 7 | 0.4±0.0 | 1.3±0.2 | -81.8 | 3.3 | 100 | 4.8 | 100:0 | 8.8 | 84.9 |
|  | Cinnamon | 7 | 0.4±0.0 | 3.1±0.4 | -55.3 | 8.1 | 100 | 5.6 | 100:0 | 8.4 | 68.6 |
|  | Wintergreen | 7 | 0.4±0.0 | 1.8±0.5 | -74.7 | 4.6 | 100 | 4.0 | 100:0 | 8.4 | 70.6 |
| White Fox | Double Mint | 12**^2^** | 0.6±0.0 | 7.8±0.8 | -35.4 | 12.7 | 100 | 8.5 | 100:0 | 8.5 | 86.9 |
| ZYN | Chill | 3 | 0.4±0.0 | 3.2±0.0 | 7.5 | 8.4 | 100 | 3.2 | 100:0 | 8.8 | 73.8 |
|  | Menthol | 3 | 0.4±0.0 | 3.4±0.1 | 13.6 | 8.4 | 94.9 | 3.2 | 100:0 | 8.3 | 68.1 |
|  | Peppermint | 3 | 0.4±0.0 | 3.2±0.0 | 7.2 | 8.0 | 93.8 | 3.0 | 100:0 | 8.3 | 67.6 |
|  | Spearmint | 3 | 0.4±0.0 | 3.2±0.1 | 6.2 | 7.7 | 83.6 | 2.7 | 100:0 | 8.5 | 75.6 |
|  | Wintergreen | 3 | 0.4±0.0 | 3.4±0.2 | 12.8 | 8.3 | 100 | 3.9 | 100:0 | 8.3 | 65.6 |
|  | Chill | 6 | 0.4±0.0 | 5.7±0.5 | -4.9 | 14.0 | 100 | 6.2 | 100:0 | 8.5 | 76.4 |
|  | Cinnamon | 6 | 0.4±0.0 | 5.7±0.2 | -4.9 | 13.2 | 100 | 6.1 | 100:0 | 8.3 | 67.1 |
|  | Citrus | 6 | 0.4±0.0 | 5.4±0.3 | -9.3 | 13.4 | 100 | 6.1 | 100:0 | 8.5 | 76.4 |
|  | Coffee | 6 | 0.5±0.0 | 6.3±0.3 | 5.5 | 13.9 | 100 | 6.3 | 100:0 | 8.5 | 74.3 |
|  | Cool Mint | 6 | 0.4±0.0 | 5.8±0.4 | -3.5 | 14.9 | 95.0 | 5.5 | 100:0 | 8.2 | 62.4 |
|  | Smooth | 6 | 0.4±0.0 | 5.4±0.1 | -9.6 | 13.7 | 100 | 6.1 | 100:0 | 8.3 | 63.5 |
|  | Wintergreen | 6 | 0.4±0.0 | 5.3±0.5 | -10.9 | 13.4 | 100 | 5.6 | 100:0 | 8.5 | 76.8 |
| **Tobacco-Containing Pouches (OTPs)** | | | | | | | | | | | |
| Bull Dog | Cold Extreme | 22**^2^** | 0.9±0.0 | 27.3±3.0 | 24.1 | 30.0 | 30.0 | 8.2 | 100:0 | 8.7 | 83.7 |
|  | Extreme | 22**^2^** | 0.9±0.1 | 25.5±2.5 | 15.9 | 29.6 | 40.1 | 10.2 | 100:0 | 8.8 | 84.9 |
| Camel | Frost | Not Listed | 0.6±0.0 | 5.4±0.3 | --- | 9.0 | 48.4 | 2.6 | 100:0 | 7.5 | 21.6 |
|  | Mellow | Not Listed | 0.6±0.0 | 6.8±0.8 | --- | 12.2 | 52.3 | 3.6 | 100:0 | 7.3 | 17.6 |
|  | Mint | Not Listed | 0.5±0.0 | 6.5±0.3 | --- | 13.5 | 63.6 | 4.1 | 100:0 | 7.3 | 16.3 |
| Copen-hagen | Wintergreen | Not Listed | 1.6±0.1 | 10.5±0.6 | --- | 6.7 | 100 | 10.7 | 100:0 | 6.8 | 5.2 |
| General | Mint | Not Listed | 0.9±0.1 | 4.9±1.3 | --- | 5.2 | 99.6 | 4.9 | 100:0 | 8.4 | 72.0 |
|  | White | Not Listed | 0.9±0.0 | 4.1±1.0 | --- | 4.5 | 92.5 | 3.8 | 100:0 | 8.4 | 70.1 |
| Grizzly | Premium Wintergreen | Not Listed | 1.3±0.0 | 11.7±1.0 | --- | 8.8 | 80.3 | 9.4 | 100:0 | 7.8 | 39.2 |
| Siberia | Mint | Not Listed | 0.7±0.0 | 19.2±2.1 | --- | 25.6 | 68.4 | 13.1 | 100:0 | 9.4 | 96.0 |
| Skoal | Classic Wintergreen | Not Listed | 1.5±0.0 | 18.2±0.4 | --- | 12.3 | 70.2 | 12.8 | 100:0 | 7.2 | 12.1 |
| **Average ONPs** | | 7.1  ±3.2 | **0.5**  **±0.1^1^** | **6.4**  **±3.5^1^** | -10.3  ±24.0 | 14.6  ±7.4 | **91.3**  **±18.5^1^** | 6.5  ±3.9 | --- | 8.3  ±0.6 | 63.4  ±25.2 |
| **Average OTPs** | | --- | **0.9**  **±0.4^1^** | **12.3**  **±8.2^1^** | --- | 14.3  ±9.6 | **67.8**  **±23.8^1^** | 7.6  ±3.9 | --- | 8.0  ±0.8 | 47.2  ±34.4 |

**^1^Indicates statistical significance (p<0.05) between ONPs and OTPs using a non-parametric Mann-Whitney t-test**

**^2^Indicates that a concentration was not listed. However, a scale was used on the package, "Paw Prints", to indicate concentration which could be compared to online claims.**

**Measured nicotine content and pH validated using CORESTA CRP1.1 (2016): Results were within 20% of previously reported values (nicotine: measured 0.68±0.03% vs reported 0.76%; pH: measured 7.62±0.07 vs reported 8.30). Bunch J, Wagner K, Morton M. CORESTA Reference Products. 2014.** [**https://www.coresta.org/sites/default/files/technical_documents/main/STS-105-CTR_2016-CRP-AnalysisWG4_Jan2017.pdf**](https://www.coresta.org/sites/default/files/technical_documents/main/STS-105-CTR_2016-CRP-AnalysisWG4_Jan2017.pdf)


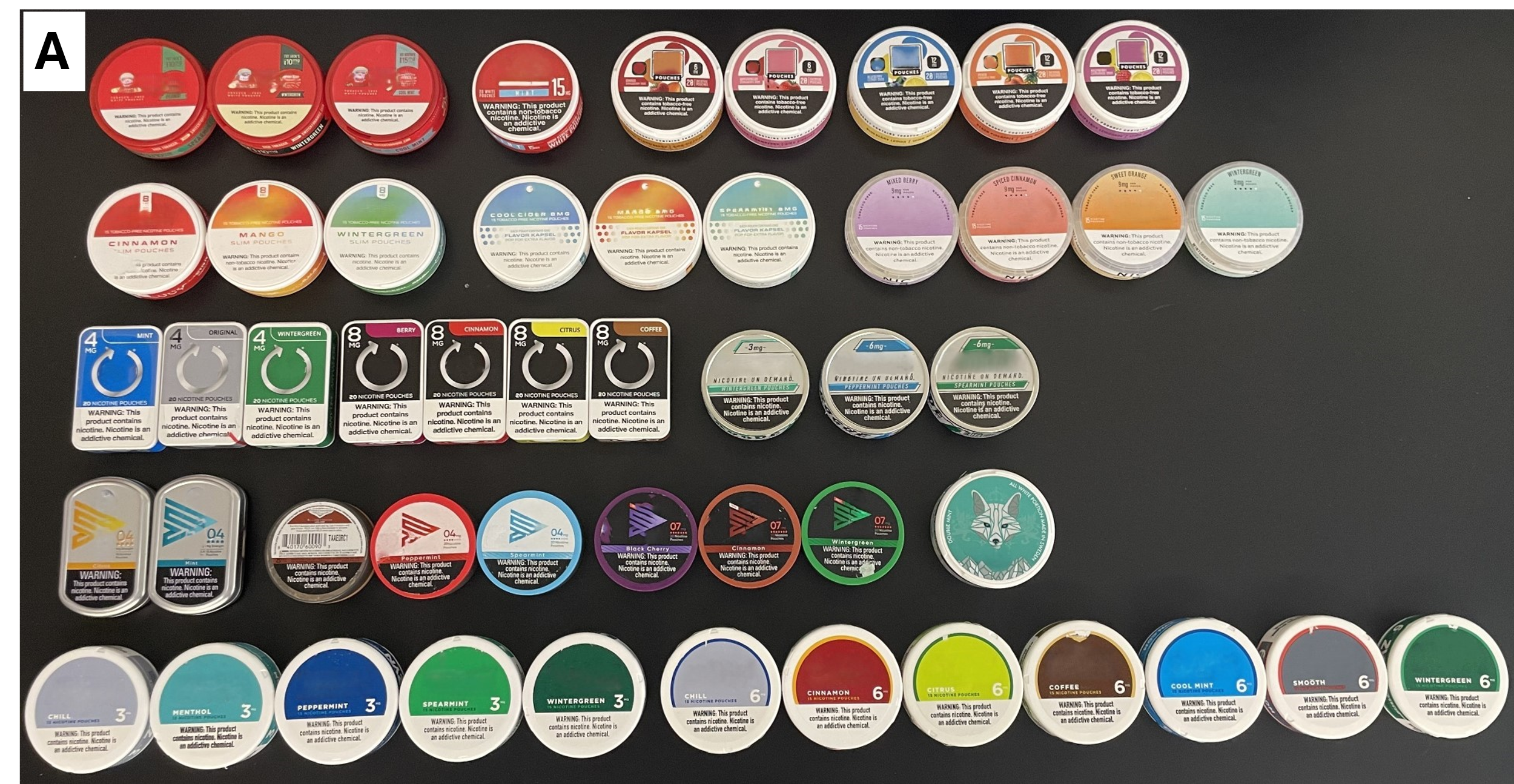


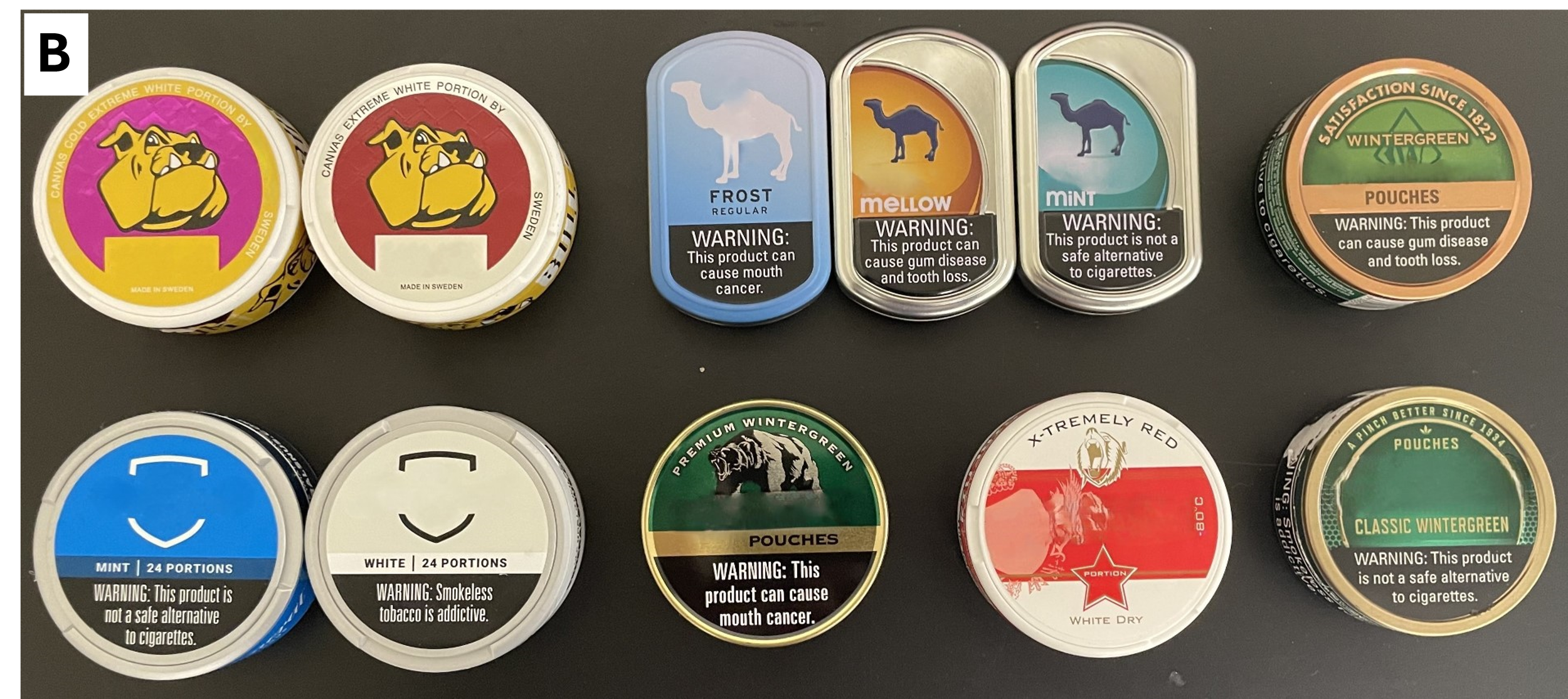


**Supplemental Figure 1.** (**A**) Pictures of oral tobacco-free pouches (ONPs) and (**B**) oral tobacco-containing pouches (OTPs) included in this study. *Indicates VELO metal tin. #Indicates VELO plastic can. Brand names have been removed using AI Generative Erase.

**Alt text:** Two images labeled A and B. A contains the packages of 50 ONP brands and flavors, where brand descriptors have been removed. B contains the packages of 11 OTP brands and flavors, where brand descriptors have been removed.

**Supplemental Table 2.** Lower limits of quantitation (LLOQ) for all chemicals quantified in this study. Nicotine release and R/S nicotine were determined using liquid chromatography/tandem mass spectrometry (LC-MS/MS), and all other analyses were completed using gas chromatography/mass spectrometry (GC/MS).

| **Chemical** | **Instrument LLOQ (µg/mL)** | **Pouch LLOQ (mg/pouch)** |
| --- | --- | --- |
| Nicotine (Content) | 1.00 | 0.20 |
| Nicotine (R/S) | 0.01 | 0.02 |
| Nicotine (Release) | 0.01 | 0.02 |
| 1,4 Cineole | 0.25 | 0.05 |
| 2,3,5-Trimethylpyrazine | 0.25 | 0.05 |
| Acetoin | 5.00 | 1.00 |
| Benzaldehyde | 0.25 | 0.05 |
| Benzyl Alcohol | 0.50 | 0.10 |
| Butanoic Acid | 50.00 | 10.00 |
| Carvone | 0.50 | 0.10 |
| Cinnamaldehyde | 10.00 | 2.00 |
| Dihydroxyacetone | 50.00 | 10.00 |
| Ethyl Maltol | 25.00 | 5.00 |
| Ethyl Salicylate | 1.00 | 0.20 |
| Ethyl Vanillin | 10.00 | 2.00 |
| Eucalyptol | 0.25 | 0.05 |
| Eugenol | 1.00 | 0.20 |
| Fenchol | 0.50 | 0.10 |
| Furaneol | 10.00 | 2.00 |
| Isomenthol | 1.00 | 0.20 |
| Isopulegol | 1.00 | 0.20 |
| Isovanillin | 25.00 | 5.00 |
| Limonene | 0.50 | 0.10 |
| Linalool | 0.50 | 0.10 |
| Maltol | 50.00 | 10.00 |
| Menthol | 0.50 | 0.10 |
| Menthone | 0.25 | 0.05 |
| Menthyl Acetate | 0.50 | 0.10 |
| Methyl Salicylate | 1.00 | 0.20 |
| Pipertone | 0.50 | 0.10 |
| Pulegone | 0.50 | 0.10 |
| Raspberry Ketone | 1.00 | 0.20 |
| Triacetin | 0.50 | 0.10 |
| Vanillin | 10.00 | 2.00 |
| WS-3 | 1.00 | 0.20 |
| WS-23 | 1.00 | 0.20 |

**Supplemental Table 3.** A summary by brand of reported contents and instructions for use for oral tobacco-free pouches (ONPs) and oral tobacco-containing pouches (OTPs) included in this study.

| **Brand** | **Nicotine Source** | **Sweeteners** | **Filler Material, Thickeners & Stabilizers** | **pH adjusters** | **Miscellaneous Claims** | **Use Duration (min)** |
| --- | --- | --- | --- | --- | --- | --- |
| Nicotine Pouches (ONPs) | | | | | | |
| BRIDGE | Non-tobacco nicotine | Sucralose | Xanthan Gum | Sodium bicarbonate | Plant fiber, water, sodium chloride, propylene glycol, flavoring | 30 |
| FRĒ | Non-tobacco nicotine | Sucralose | Xanthan Gum | Sodium bicarbonate | Plant fiber, water, sodium chloride, flavoring | 30 |
| Juice Head | Zero-tobacco synthetic nicotine | Acesulfame potassium Sucralose | Cellulose Sodium alginate Xylitol | Sodium bicarbonate | Salt, glycol, propylene glycol, potassium sorbate, flavoring | 10-30 |
| LUCY  (Non-Capsule) | Non-tobacco nicotine | Acesulfame potassium Maltitol | Microcrystalline cellulose Polacrilex | Sodium carbonate | Calcium lactate, MCT, glycerol, propylene glycol, sugar-free, food grade flavoring | Up to 45 |
| LUCY "Kapsel" | Non-tobacco nicotine | Erythritol Sucralose | Microcrystalline cellulose Hydroxypropyl cellulose Polacrilex | None identified | Calcium lactate, MCT, propylene glycol potassium sorbate, sugar-free, gelatin, food grade flavoring | Up to 45 |
| NIC-S | Non-tobacco derived nicotine | None identified | Polacrilex | None identified | Water, flavoring | 15-30 |
| on! | Nicotine salt (derived from tobacco) | None identified | Microcrystalline cellulose | Sodium carbonate | Binders, flavoring | 20 |
| Rogue | Tobacco-leaf free | Acesulfame potassium | Polacrilex | None identified | Sugar & gluten free, inactive food grade ingredients for flavor and texture | Up to 60 |
| VELO (Tin) | Extracted from tobacco | Sucralose | Microcrystalline cellulose | None identified | Water, salt, citric acid, artificial flavor | Up to 30 |
| VELO (Can) | Extracted from tobacco | Acesulfame potassium Maltitol | Modified cellulose | Sodium carbonate | Artificial flavor | Up to 30 |
| White Fox | None identified | None identified | Microcrystalline cellulose  Xanthan gum | Sodium bicarbonate | Natural aroma oils, water, salt, vegetable oil, propylene glycol | None identified |
| ZYN | Nicotine salt (nicotine bitartrate dihydrate) | Acesulfame potassium | Microcrystalline cellulose Maltitol Gum arabic Hydroxypropyl cellulose | Sodium bicarbonate Sodium carbonate | Food grade flavorings Lactose-free, gluten-free (<20 ppm), <1% daily recommended carbohydrates, <10 mg sodium (<0.5% daily recommended value) | Up to 60 |
| Tobacco Pouches (OTPs) | | | | | | |
| Bull Dog | None identified | None identified | Tobacco Microcrystalline cellulose Xanthan gum | Potassium carbonate | Water, salt, propylene glycol, natural oils, natural flavoring | None identified |
| Camel | 100% imported tobacco | Sucralose | Tobacco Pouch materials | Sodium bicarbonate Sodium carbonate | Water, sodium chloride, propylene glycol, natural & artificial flavoring | Up to 30 |
| Copenhagen | 100% American tobacco | Sucralose Sodium saccharin | Tobacco Pouch materials | Ammonium carbonate Sodium carbonate | Binders, water, sodium chloride, ethyl alcohol, preservatives, natural & artificial flavorings | None identified |
| General | 100% premium imported tobacco | Acesulfame potassium | Tobacco | Sodium carbonate Magnesium carbonate | Water, table salt, propylene glycol, natural & artificial flavoring incl. artificial smoke flavor | Up to 30 |
| Grizzly | 70% American tobacco 30% Brazilian tobacco | None identified | Tobacco | None identified | None identified | None identified |
| Siberia | None identified | None identified | Tobacco | Potassium carbonate | Water, salt, propylene glycol; natural oils | 30-45 |
| Skoal | 100% American tobacco | Sucralose Sodium saccharin | Tobacco Pouch materials | Ammonium carbonate Sodium carbonate | Binders, water, sodium chloride, ethyl alcohol, preservatives, natural & artificial flavorings | None identified |

**Supplemental Table 4.** A summary of warning labels by brand found on packaging for all oral tobacco-free pouches (ONPs) and oral tobacco-containing pouches (OTPs) included in this study.

| **Label Category** | **Frequency of Products** | |
| --- | --- | --- |
|  | **ONPs  n (%)** | **OTPs  n (%)** |
| **Health Warning** |  |  |
| Damages to Health: Mouth Cancer | 0 (0%) | 2 (29%) |
| Damages to Health: Gum Disease | 0 (0%) | 4 (57%) |
| Damages to Health: Tooth Loss | 0 (0%) | 4 (57%) |
| Damages to Health: Non-Specific | 0 (0%) | 2 (29%) |
| California Proposition 65 | 7 (70%) | 0 (0%) |
| Product is Addictive | 0 (0%) | 4 (57%) |
|  |  |  |
| **Nicotine Content** |  |  |
| Contains Nicotine | 10 (100%) | 0 (0%) |
| Nicotine is Addictive | 10 (100%) | 0 (0%) |
|  |  |  |
| **Legal Disclaimers** |  |  |
| Not a Safe Alternative to Cigarettes | 0 (0%) | 4 (57%) |


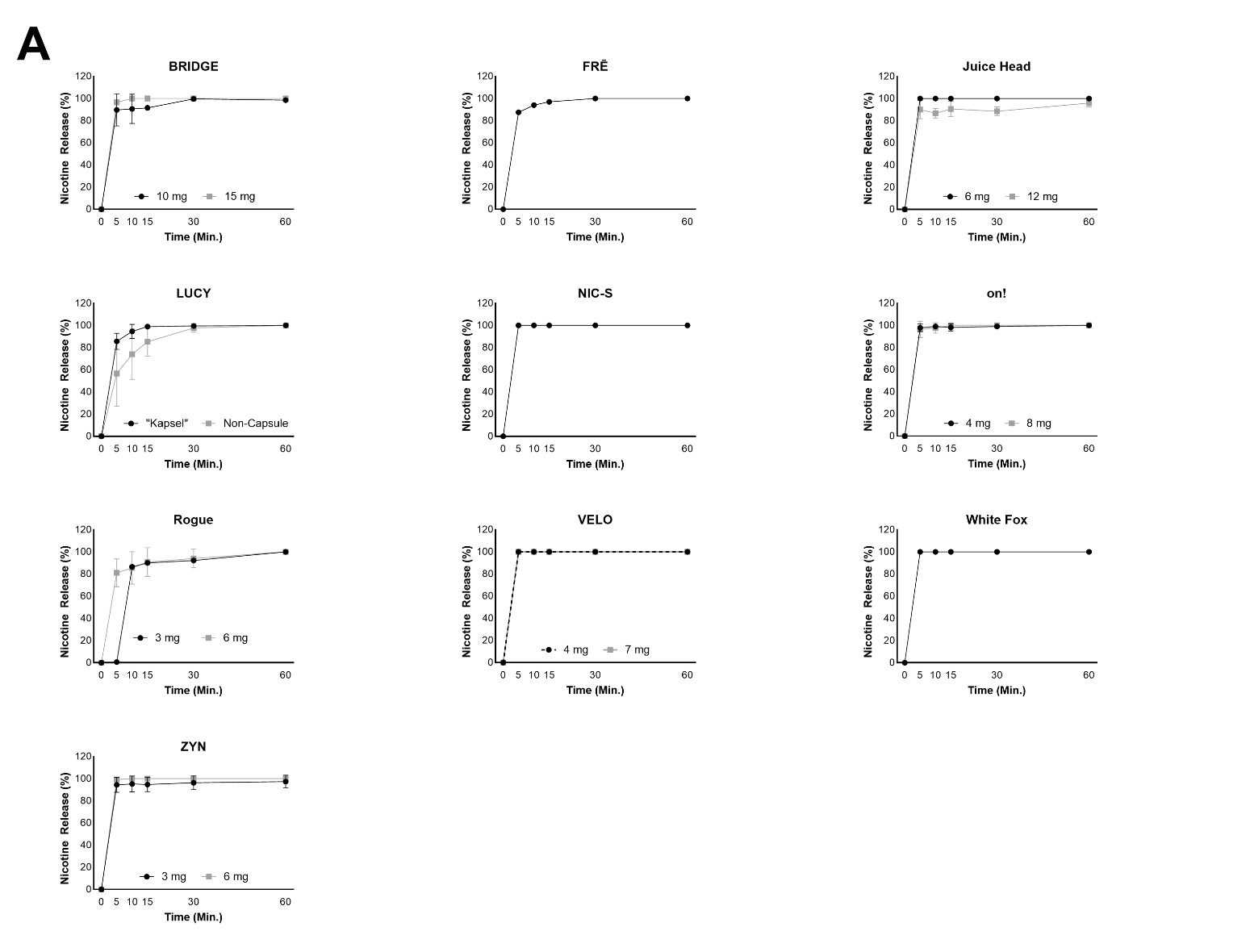

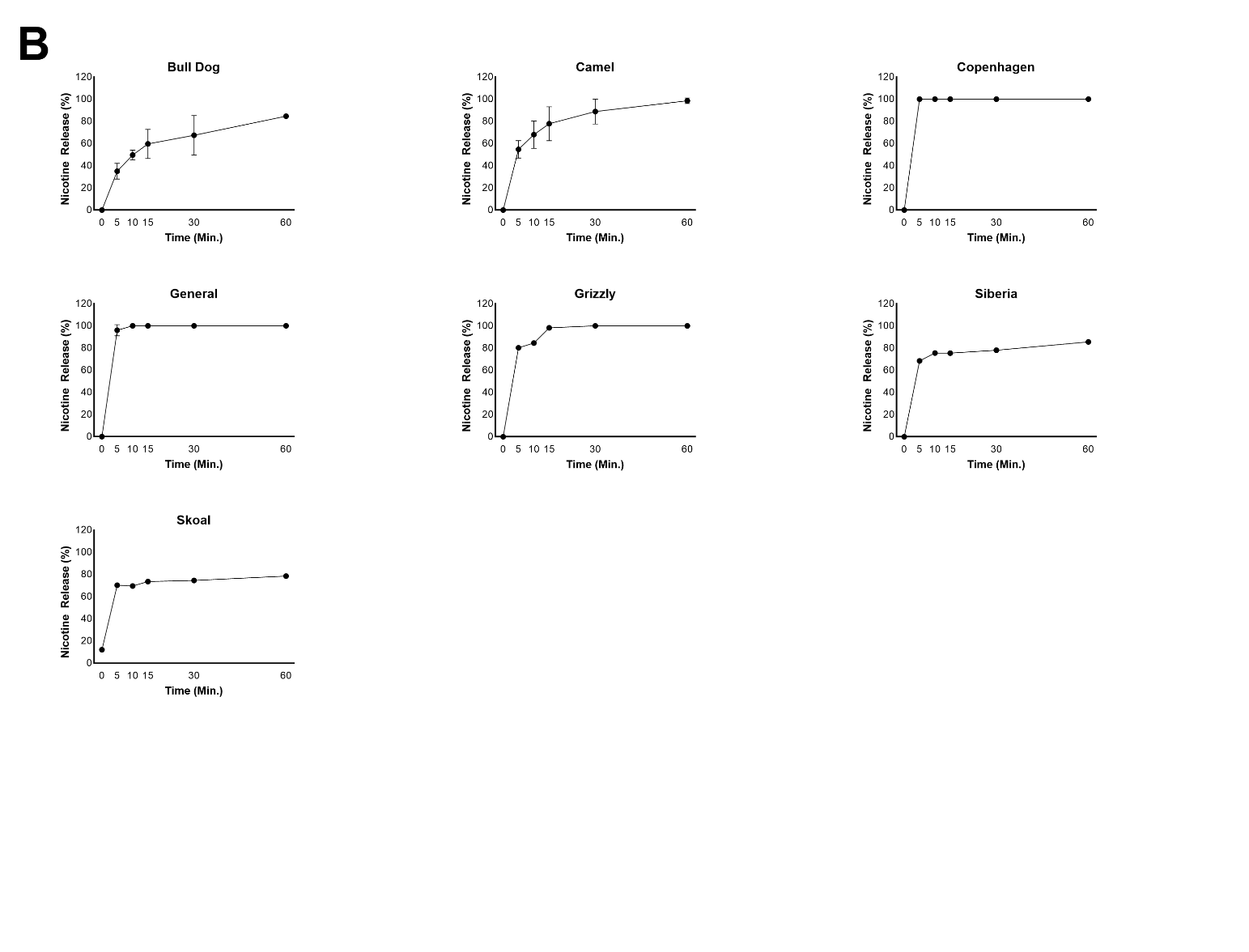


**Supplemental Figure 2.** (**A**) The percentage of nicotine release by brand and reported nicotine concentration for individual brands of oral tobacco-free pouches (ONPs) and (**B**) oral tobacco-containing pouches (OTPs) are included in this study. Error bars represent standard deviation.

**Alt text:** Images of nicotine released from individual ONP brands, labeled as A, or individual OTP brands, labeled as B, at 5, 10, 15, 30 and 60 minutes.

**Supplemental Table 5.** A summary of flavoring chemicals by flavor category found in oral tobacco-free pouches (ONPs) and oral tobacco-containing pouches (OTPs) included in this study.

| **Flavor Category (Krüsemann et al., 2018)** | **Flavor Subcategory** | **Tobacco-Free Pouches**  **(ONPs)** | |  | **Tobacco-Containing Pouches (OTPs)** | |
| --- | --- | --- | --- | --- | --- | --- |
|  |  | **Number of Brands** | **Identified Flavorings (number of products)** |  | **Number of Brands** | **Identified Flavorings**  **(number of products)** |
| Coffee | Coffee | 3 | None detected |  | --- | --- |
| Fruit | Berry | 3 | Menthol (1) Benzyl alcohol (1) |  | --- | --- |
|  | Citrus | 4 | Menthol (2) Limonene (1) |  | --- | --- |
|  | Mango | 2 | Benzyl alcohol (2) Limonene (1) Menthol (1) Triacetin (1) |  | --- | --- |
| Hybrid | Beverage | 2 | Menthol (2) Benzyl alcohol (1) Eucalyptol (1) Limonene (1) Menthone (1)  Pulegone (1) Triacetin (1) Raspberry ketone (1) WS-23 (1) |  | --- | --- |
|  | Mixed Fruit | 4 | Menthol (4) Benzyl alcohol (2) |  | --- | --- |
| Mint/Cooling | Menthol | 1 | Eucalyptol (1) Menthol (1) |  | --- | --- |
|  | Mint | 6 | Menthol (6) Menthone (4) Menthyl acetate (4) Carvone (1) Eucalyptol (1) |  | 3 | Menthol (3) Eucalyptol (2) Menthone (2) Menthyl acetate (2) WS-3 (2) Benzyl alcohol (1) Carvone (1) Limonene (1) Piperitone (1) WS-23 (1) |
|  | Peppermint | 3 | Menthol (3) Menthone (3) Menthyl acetate (1) |  | --- | --- |
|  | Spearmint | 5 | Menthol (4) Menthone (3) Carvone (3) Limonene (2) Benzyl alcohol (1) Eucalyptol (1) Menthyl acetate (1) Pulegone (1) WS-3 (1) |  | --- | --- |
|  | Wintergreen | 8 | Methyl salicylate (8) Menthol (5) Menthone (2) Benzyl Alcohol (2) Triacetin (1) |  | 3 | Methyl salicylate (3) Menthol (1) |
|  | Unknown (e.g., Chill, Cold, Frost) | 2 | WS-3 (2) |  | 2 | Benzyl Alcohol (1) Carvone (1) Menthol (1) WS-3 (1) WS-23 (1) |
| Spice | Cinnamon | 5 | Cinnamaldehyde (2) Benzaldehyde (1) Benzyl alcohol (1) Menthol (1) |  | --- | --- |
| Unknown | Unknown (e.g., Extreme, Mellow, Original, Smooth, White) | 2 | None detected |  | 3 | Benzyl alcohol (1) Linalool (1) |

Krüsemann, Erna JZ, et al. An e-liquid flavor wheel: a shared vocabulary based on systematically reviewing e-liquid flavor classifications in literature. *Nicotine and Tobacco Research. 2019;* 21.10: 1310-1319.
